# Dilution of Seawater Affects the Ca^2 +^ Transport in the Outer Mantle Epithelium of *Crassostrea gigas*

**DOI:** 10.3389/fphys.2020.00001

**Published:** 2020-01-22

**Authors:** J. Kirsikka Sillanpää, Joao Carlos dos Reis Cardoso, Rute Castelo Félix, Liliana Anjos, Deborah Mary Power, Kristina Sundell

**Affiliations:** ^1^Fish Endocrinology Laboratory, Department of Biological and Environmental Sciences, University of Gothenburg, Gothenburg, Sweden; ^2^Comparative Endocrinology and Integrative Biology, Centre of Marine Sciences, Universidade do Algarve, Faro, Portugal

**Keywords:** Ca^2+^-ATPase, Na^+^/Ca^2+^-exchanger, calcium channel, Na^+^/K^+^-ATPase, calcification, salinity

## Abstract

Varying salinities of coastal waters are likely to affect the physiology and ion transport capabilities of calcifying marine organisms such as bivalves. To investigate the physiological effect of decreased environmental salinity in bivalves, adult oysters (*Crassostrea gigas*) were exposed for 14 days to 50% seawater (14) and the effects on mantle ion transport, electrophysiology and the expression of Ca^2+^ transporters and channels relative to animals maintained in full strength sea water (28) was evaluated. Exposure of oysters to a salinity of 14 decreased the active mantle transepithelial ion transport and specifically affected Ca^2+^ transfer. Gene expression of the Na^+^/K^+^-ATPase and the sarco(endo)plasmic reticulum Ca^2+^-ATPase was decreased whereas the expression of the T-type voltage-gated Ca channel and the Na^+^/Ca^2+^-exchanger increased compared to animals maintained in full SW. The results indicate that decreased environmental salinities will most likely affect not only osmoregulation but also bivalve biomineralization and shell formation.

## Introduction

Intertidal organisms face a fluctuating environment where the salinity can exhibit drastic changes during a single day (Geng et al., 2016). These natural variations in salinity may be further accentuated by the changes occurring in oceans due to climate change, which is expected to modify water evaporation and precipitation patterns in the near future (Ipcc, 2014). Many marine osmoconformers lack the capacity to regulate osmolarity and the ion content of their internal fluids, especially the extracellular fluids that are in equilibrium with their environment making them highly susceptible to salinity fluctuations. Marine calcifiers need Ca^2+^ and CO_3_^2–^ ions to build their shells. Part of the required CO_3_^2–^ is produced metabolically and the Ca^2+^ is taken up from the environment or from food (Sillanpää et al., 2016; Zhao et al., 2018b). Therefore changes in seawater salinity and thus ion concentrations is likely to affect the physiology of marine calcifying organisms. Salinity stress has been shown to trigger multiple physiological responses in marine calcifying invertebrates including changes in protein and mRNA expression, enzyme metabolism, cell signaling, amino acid content and ion regulation (Shumway, 1977a; Berger and Kharazova, 1997; Zhao et al., 2012; Meng et al., 2013). A decrease in environmental water salinity was shown to decrease shell calcification and/or affect shell properties in species such as *Crassostrea virginica* and *Mytilus edulis* (Malone and Dodd, 1967; Dickinson et al., 2012; Sanders et al., 2018) and alter the expression and function of nacrein, a carbonic anhydrase enzyme with a key role in bivalve shell formation in *Mytilus galloprovincialis* (Cardoso et al., 2019). Nonetheless, it remains unclear how environmental salinity, ion regulation mechanisms and calcification are interconnected.

Bivalves are a class of mollusks that have laterally compressed bodies enclosed in a calcium carbonate (CaCO_3_) shell, which offers protection from the environment and gives support to the animal body. The mantle tissue, which covers the somatic mass and lines the shell is the main shell producing organ in bivalves (Marin et al., 2012; Xiang et al., 2014). The tissue organization of the bivalve mantle is conserved. It is composed of an inner layer of muscles, nerve fibers and connective tissue covered on either side by epithelial cells (Marin et al., 2012). The outer mantle epithelium (OME) is the shell-facing epithelial layer, which transports and secretes shell components. Calcification of the shell occurs in the extrapallial space (EPS) between the OME and shell (Wheeler and Sikes, 1984; Marin et al., 2012).

The mantle is a heterogeneous organ and the different mantle zones vary in morphology and function (Gardner et al., 2011; Bjärnmark et al., 2016). Traditionally, the bivalve mantle is divided into three zones: the central, the pallial, and the mantle edge (Kádár, 2008; Bjärnmark et al., 2016). The differences in morphology between the different mantle zones have also been suggested to reflect their functions, such as ion transporting abilities. The mantle edge, consisting of three separate folds, the inner, the middle, and the outer fold, is suggested to be especially active in calcification (Kádár, 2008). However, the pallial mantle zone has also been shown to actively transport Ca^2+^ (Sillanpää et al., 2018). In addition to transporting Ca^2+^, cultured mantle cells have been observed to precipitate CaCO_3_ intracellularly (Xiang et al., 2014). The mantle is also the main organ responsible for secreting the organic matrix onto which CaCO_3_ precipitates (Wheeler and Sikes, 1984; Wang et al., 2013). The proteins making up the shell matrix control the location and timing of the biomineralization process as well as the shape and orientation of the minerals (Marin and Luquet, 2004; Takeuchi and Endo, 2006; Gardner et al., 2011).

A constant supply of calcium and carbonate ions is essential for bivalves to build and maintain their shell. Calcium is mostly taken up across the gills, mantle tissue and the gut as ionic calcium (Ca^2+^) and is then transferred via the hemolymph to the EPS (Rousseau et al., 2003; Fan et al., 2007b; Sillanpää et al., 2016). Hemolymph ion concentrations of marine bivalves deviates only slightly from the environmental ion concentrations (Shumway, 1977a; Knowles et al., 2014; Thomsen et al., 2015; Sillanpää et al., 2016). Multiple pathways and mechanisms have been suggested for the transfer of calcium across the OME. The movement of ionic Ca^2+^ across the OME may be passive or through Ca^2+^ transporting membrane-proteins and channels (Ip et al., 2017; Sillanpää et al., 2018). Alternatively, Ca^2+^ could be transferred bound to Ca-binding proteins through mantle epithelial cells (Nair and Robinson, 1998; Xue et al., 2012). Specialized hemocytes or intracellular vesicles containing CaCO_3_ crystals have also been proposed to participate in the transfer of calcium to the EPS (Mount et al., 2004; Cho and Jeong, 2011; Li et al., 2016).

The transfer of Ca^2+^ across the OME of the Pacific oyster, *Crassostrea gigas* involves plasma-membrane Ca^2+^-ATPases (PMCA), voltage-gated Ca channels (VGCC) and potentially Na^+^/Ca^2+^ -exchangers (NCX) together with passive paracellular ion transfer, between the OME cells (Sillanpää et al., 2018). PMCA and NCX seem to have almost equal importance in the transfer of Ca^2+^ across the OME, and each contributes approximately 30% to the total transfer (Sillanpää et al., 2018). Genome sequencing of *C. gigas*, has revealed a variety of PMCA, sarco(endo)plasmic reticulum Ca^2+^-ATPase (SERCA) or L-type calcium channel transcripts (Zhang et al., 2012) whereas no genes annotated as NCX were found. Transcripts for PMCA, NCX, and/or Ca channels have also been identified in other bivalve species such as *Pinctada fucata*, *M. edulis*, and *Tridacna squamosa* (Fan et al., 2007b; Wang et al., 2008; Ip et al., 2017, Ramesh et al., 2019) suggesting common transport systems are conserved across different species. Similarly, carbonate, either in the form of HCO_3_^–^ or CO_3_^2–^, has been suggested to be transported through active transporters such as the Cl^–^/HCO_3_^–^ exchanger to the shell growth area (Zhao et al., 2018a; Ramesh et al., 2019).

In the present study, we aimed to investigate the functional model we previously proposed, in which PMCA, NCX, and VGCCs in the Pacific oyster OME most likely participate in transferring Ca^2+^ to the shell growth area (Sillanpää et al., 2018). Furthermore, the model is extended in the present study to investigate how lowering of environmental salinity is likely to impact the Ca transporting mechanisms of the OME of *C. gigas*.

## Materials and Methods

### Experimental Set-Up

**FIGURE 1:**
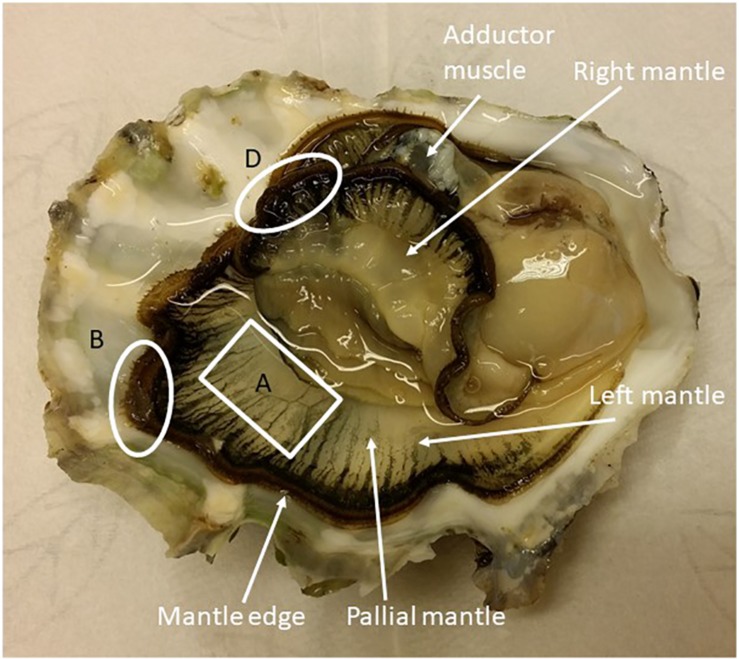
*C. gigas* interior with the two mantle halves exposed. Sampling for Ussing chamber experiments were taken from area A (pallial mantle) and for qPCR analysis from areas B and D (mantle edge).

Adult Pacific oysters, *C. gigas* with a mean weight of 111.9 ± 2.07 g (mean ± SEM) and length of 10.8 ± 0.14 cm were purchased from Huîtres Baudit (Charente Maritime, France). They were transferred to the aquaria facilities at the Department of Biological and Environmental Sciences, University of Gothenburg, Sweden and acclimated to recirculating artificial seawater (Aquaforest, Poland) at 10°C, for at least 1 week prior to the start of experiments. The oysters were randomly divided into eight identical 15 L plastic containers, 5 oysters/container, filled with 10 L of water from the recirculating system used for acclimation. Half of the containers were filled with seawater with a salinity of 28 (osmolality 805 ± 16 mOsm ⋅ kg^–1^ and [Ca] 6.06 ± 0.22 mM) and the other containers with seawater diluted to 50% with tap water to give a final salinity of 14 (osmolality 362 ± 11 mOsm ⋅ kg^–1^ and [Ca] 3.17 ± 0.10 mM). The water was oxygenated using compressed air, which also maintained the water pH at 8.11 ± 0.03 for the duration (14 days) of the experiment. The oysters were not fed during the experiment, and the water was exchanged every other day to avoid accumulation of metabolic waste and feces. After 14 days of exposure to full strength seawater (28) or 50% SW (14), 4 oysters from each experimental tank were sampled for Ussing chamber experiments and two pieces of the mantle were removed for quantitative PCR (qPCR) analysis (Figure 1, see sections “*In silico* Analyses” and “qPCR Analysis”). Because only eight samples can be simultaneously analyzed with the Ussing chamber set-up available, the start of the experiment and the sampling were executed on two consecutive days (16 oysters/day). Mortality during the experiment was 10% (salinity 28: 5%, 14: 15%).

### Sampling

Oysters were removed from the tanks, dried with paper and then the valves were pried open from the umbo. Hemolymph was withdrawn from the adductor muscle using a 60 mm 23 gauge needle affixed to a sterile 1 ml plastic syringe. After sampling the hemolymph, the shell was fully opened and around 20–25 mg of tissue was collected from the left and right mantle edge for RNA extraction (Figure 1). The mantle tissue samples were washed with fresh Ringer solution and snap-frozen in liquid nitrogen. For physiological measurement of Ca^2+^ transport and the electrical characteristics of the OME a piece of the left ventral pallial mantle, approximately 2 × 4 cm (Figure 1), was dissected out. The inner mantle epithelium and connective tissue were carefully removed by blunt dissection and the remaining OME was mounted into modified Ussing chambers (Sundell et al., 2003; Sillanpää et al., 2018). The hemolymph samples were analyzed for [Ca^2+^], [Na^+^], [K^+^], and [Cl^–^] with an Ion Electrolyte Analyzer (Convergys ISE Comfort, Convergent Technologies, Germany).

### Functional Analyses Using Ussing Chamber Methodology

The Ussing chamber methodology was used to study the Ca^2+^ transfer across the OME and the electrophysiology of the OME of *C. gigas*. Transfer rates across the OME of radiolabelled marker molecules, ^45^Ca and ^3^H-mannitol, were assessed in custom-built modified Ussing chambers of the Grass/Sweetana type (Grass and Sweetana, 1988) with an opening of 0.75 cm^2^ (Sundell et al., 2003). An electronic Ussing chamber control and measurement system, UCC-401 (UCC-Labs Ltd., Gothenburg, Sweden) was used to assess the electrical parameters, transepithelial resistance (TER), transepithelial potential (TEP), and short circuit current (SCC), as previously described in Sundell and Sundh (2012) and Sillanpää et al. (2018). TEP values are referenced to the hemolymph side of the OME preparation.

After mounting the OMEs in the Ussing chambers, both half-chambers were filled with 4 ml of ice-cooled oyster Ringer solution (NaCl 447 mM, KCl 14.5 mM, MgSO_4_ 12.9 mM, MgCl_2_ 10.6 mM, CaCl_2_ 10 mM, NaHCO_3_ 3.6 mM, NaHPO_4_ 0.3 mM, glucose 0.3 mg/ml, 811 ± 41 mOsm ⋅ kg^–1^). The mixing of the Ringer solution within the half chambers and the maintenance of constant oxygen and pH (7.33 ± 0.03; Metrohm 744 pH meter, Switzerland) levels was achieved by constant bubbling with a gas-air mixture containing 0.3% CO_2_. The mantle tissues were allowed to stabilize for 60 min after which the Ringer solution was exchanged. 4 ml of fresh Ringer solution was added to the right half chamber (shell side of the OME), while 4 ml of Ringer solution containing 0.015 MBq radiolabelled ^45^Ca (Specific activity 458.65 MBq/mg PerkinElmer, MA, United States) and 0.56 MBq ^3^H labeled mannitol were applied to the left half chamber (the “hemolymph” side of the OME). Samples of 50 μl were immediately taken from both half chambers to assess the amount of ^45^Ca at the starting point of the experiment. To assess the rate of ^45^Ca transfer as well as the appearance rate of ^3^H-mannitol (an inert hydrophilic molecule used to assess the paracellular permeability of the OME), 50 μl samples were taken from the “shell” side half chamber at 10, 15, 20, 60, 80, 85, and 90 min after changing the Ringer solution. Radioactivity of the samples was counted after addition of 4.5 ml of scintillation fluid Ultima Gold (PerkinElmer, Waltham, MA, United States) using a liquid scintillation counter (Wallac 1409 Liquid Scintillation β-Counter, Turku, Finland) with a dual label protocol for ^3^H and ^45^Ca. TER, TEP, and SCC were determined for the duration of the experiment (150 min) to monitor the tissue viability as well as the electrical OME permeability (TER) and active ion transport (SCC).

### Calculations

#### Half-Life Corrections

The disintegrations per minute (DPM) for ^45^Ca, were corrected for radioactive decay by calculating the fraction of remaining activity using Eq. 1 (Kahl et al., 2012).

(1) $$\mathrm{Fraction}\,\mathrm{Remaining}={\text{e}}^{\left(-0.693\,/\,t_{\text{1/2}}\right)\times \text{time}}$$

where *t*_1__/__2_ is the half-life of the isotope (162.7 day for ^45^Ca), and time (the number of days after the manufacturers reference date).

#### Transfer Rate of Ca^2+^ Across the OME

The starting concentration of the radioisotope in the hemolymph half chamber (DPM ⋅ l^–1^) was divided by the concentration of Ca^2+^ (10 mM) in the Ringer solution to give the specific activity in DPM ⋅ (mmol Ca^2+^)^–1^.

The amount of DPM from the ^45^Ca accumulated in the shell half chamber at each time point was multiplied by 80 to achieve the total DPM in the 4 ml volume. The total amount of Ca^2+^ (mM) transferred across the OME was then calculated from the total DPM using the specific activity according to Eq. 2.

(2) $$\mathrm{Ca}\,\left(\mathrm{mM}\right)=\frac{\mathrm{total}\,\mathrm{DPM}\,\mathrm{per}\,\mathrm{halfchamber}\,\left(4\,\mathrm{ml}\right)}{\text{DPM}\times {\left(\mathrm{mmol}\,{\mathrm{Ca}}^{2+}\right)}^{-1}}$$

The slope of the Ca^2+^ accumulation across 90 min was determined and the transport rate was expressed as nM ⋅ min^–1^.

The permeability of the paracellular pathways was described by the apparent permeability (*P*_app_) of ^3^H-mannitol across the OME, which was calculated using Eq. 3

(3) $$P_{\text{app}}=\frac{\text{dQ}\times {\text{dt}}^{-1}}{A_{\text{c}}\times C_{0}}$$

in which dQ ⋅ dt^–1^ describes the appearance of ^3^H-mannitol on the shell side (mol ⋅ s^–1^), *A*_*c*_ the surface area of the chamber opening (0.75 cm^2^) and *C*_0_ the initial concentration of ^3^H-mannitol on the hemolymph side (mol ⋅ ml^–1^).

### *In silico* Analyses

#### Database Sequence Searches

Putative ion-transporter genes were obtained from the annotated genome of *C. gigas* (Zhang et al., 2012): NKA subunit alpha (K1R0L4), three Ca^2+^-ATPases (K1QA13, K1PY00, K1QZU8) and three different voltage-dependent calcium channels: L-type (K1Q7D2, K1QEL8), T-type (K1RRY9) and type A subunit α-1 (K1R285). NXC (A0A0L8HMV4) from the *Octopus bimaculoides* was retrieved since it was not identified in the annotated genome of *C. gigas*. The sequences retrieved from the *C. gigas* genome as well as the *O. bimaculoides* NCX were used to search the assembled *C. gigas* genome available from Ensembl Metazoan Genomes^1^ to identify additional family members. For comparative purposes and to study gene family evolution, homologs of the putative ion-transporter genes from *C. gigas* were procured in other mollusks (bivalves, gastropods and cephalopods) as well as in annelids and a brachiopod, the lamp shell (*Lingula anatina*). The BLAST algorithm from the Ensembl Metazoan Genomes and NCBI^2^ databases (February 2018) was used to search for sequence homologs. Searches at NCBI were performed in the sub-dataset for bivalves (taxid:6544). Homologous sequences from the Mediterranean mussel (*M. galloprovincialis*) were obtained by searching available mantle transcriptomes (SRP063654) (Bjärnmark et al., 2016) using the same strategy as outlined above. Genome data for a gastropod, the owl limpet (*Lottia gigantea*), a cephalopod, the California two-spot octopus (*O. bimaculoides*), for two annelids, a leech (*Helobdella robusta*) and a worm (*Capitella teleta*) and for a brachiopod, the lamp shell (*L. anatina*) were retrieved from Ensembl Metazoan Genomes. For comparative purposes homologs from two other protostomes, a fruit fly (*Drosophila melanogaster*) and a nematode (*Caenorhabditis elegans*), from an invertebrate deuterostome, the ascidian (*Ciona intestinalis*) and from two vertebrates, the human (*Homo sapiens*) and a teleost, the zebrafish (*Danio rerio*) were also procured and retrieved from Ensembl^3^ using sequence similarity searches or genome annotations. To better understand gene evolution, two cnidarians (basal metazoans) available from NCBI (see text footnote 2, cnidarians: taxid:6073) and JGI^4^ databases were also searched for homologs of putative ion-transporter genes using the BLASTP algorithm. The identity of the retrieved sequences was confirmed by searching against the human protein sub-set database available from NCBI (taxid:9606).

#### Sequence Alignments and Phylogenetic Analysis

Multiple sequence alignments (MSA) of the deduced proteins, SERCA, PMCA, NKA, and NCX were performed using the MUSCLE algorithm (Edgar, 2004) available from the Aliview platform 1.18 (Larsson, 2014). Partial sequences were retrieved from some species and were concatenated and aligned with the full-length homologs from related species. The final MSA dataset for each gene family was used for construction of phylogenetic trees using the ML and BI methods. ML trees were carried out using PhyML 3.0 (Guindon et al., 2010) in the ATGC bioinformatics platform with SMS automatic model selection (Lefort et al., 2017) based on AIC (Akaike Information Criterion) to best study protein evolution. ML trees were constructed using an LG substitution model (Le and Gascuel, 2008) for SERCA, PMCA, NCX, and NKA and a VT model (Müller and Vingron, 2000) for the L-type and T-type Ca channel. Reliability of internal branching in phylogenetic trees was assessed using 100 bootstrap replicates. The BI tree was performed in MrBayes 3.2 (Ronquist et al., 2012) using as appropriate an LG or VT substitution models (Aamodel = LG or Aamodel = VT) and 1,000,000 generations sampling probability values to support tree branching. ML and BI trees were displayed with FigTree 1.4.2 and edited in Inkscape.

### RNA Extraction and cDNA Synthesis

The mantle tissue samples for gene expression analysis were homogenized using a Tissuelyser II (Qiagen, Germany) and total RNA was extracted using a Qiagen RNeasy Plus Mini (Germany) kit according to the manufacturer instructions. Total RNA was eluted in 30 μl RNAase free water. The quality and quantity of the isolated RNA was assessed using a NanoDrop 2000c Spectrophotometer (Thermo Fisher Scientific, Waltham, MA, United States) and the quality of 10% of the samples was also checked using an Agilent RNA 6000 Nano Kit in Agilent 2100 Bioanalyzer (CA, United States). Oyster cDNA was synthetized using an iScript cDNA Synthesis Kit (Bio-Rad). The final reaction volume was 20 μl and according to the yield of RNA extractions either 500 ng or 1000 ng of total RNA was used and iScript Reverse Transcriptase, iScript Reaction Mix and nuclease-free water was added following the manufacturer’s instructions. cDNA was synthetized for 5 min at 25°C, 30 min at 42°C, and 5 min at 85°C.

### qPCR Analysis

Real-time qPCR analysis was used to assess if exposure to decreased environmental salinity (14) affected the mRNA expression of oyster Ca^2+^ ion transporters and channels. These genes were selected based on the results from Sillanpää et al. (2018) and using the sequences retrieved from the *C. gigas* genome (see section “*In silico* Analyses”). Specific primers were designed for each gene (Table 1; Untergasser et al., 2012) and qPCR analysis and qPCR reactions were performed in duplicate (<5% variation between replicates) using a Bio-Rad CFX Connect Real Time System and SSO Advanced Universal SYBR Green kit (Bio-Rad). PCR reactions were performed in low volume 96-well microplates (Thermo Scientific, Denmark) and had a final volume of 10 μl and contained 5 ng cDNA, 0.5 μM of both forward and reverse primers and 5 μl SSO Advanced SYBR Green supermix. Optimized cycling conditions consisted of an initial 3 min at 95°C followed by 40 cycles of 10 s at 95°C and 30 s at the optimized annealing temperature for primers (Table 1). Melting curves were performed to detect non-specific products and primer dimers and control reactions were included in all runs to confirm the absence of genomic DNA contamination. Elongation factor 1-alpha (Ef1α) and GADPH were used as reference genes and were selected based on their stable expression in the samples analyzed. Transcript values were normalized against the geometric mean of the two reference genes.

**TABLE 1:** Primer sequences, annealing temperatures, *R*^2^ values and qPCR reaction efficiencies used for qPCR measurements of the candidate and reference genes.

**Gene**	**Primer sequence 5′–3′**	**T°A°**	**Efficiency**	***R*^2^**
NKA	Fwd: GGGAGGTCTTGGAGAACGTG	62	100%	0.998
	Rev: GACAGCATCTGGGACAGCAG			
PMCA	Fwd: ACGTTGGAGGATCTGGAGGA	62	101.8%	0.998
	Rev: TCAAATGCCGTGGTTAATCG			
SERCA	Fwd: TGGCAGGAGAGAAATGCTGA	62	101.5%	0.994
	Rev: GTGTGGTGGAATGGATGGTG			
NCX	Fwd: AGGAGAGGCTCAGGGAGAAG	60	99%	0.992
	Rev: CACATTGGCTTTCTTCAGCA			
L-type VGCC	Fwd: GGAGGACATGGAGGATGAGG	62	100.9%	0.998
	Rev: TGTGGCAGATGATTCGGAAC			
T-type VGCC	Fwd: AATGGCATGGAAACCACCTC	62	102%	1
	Rev: GAGGCTCTGATGCTCCCTGT			
Ef1α	Fwd: GAAGGCTGAGCGTGAACGT	56	113.6%	0.915
	Rev: TCCTGGGGCATCAATAATG			
GADPH	Fwd: GGAGACAAGCGAAGCAGCAT	60	103.6%	0.981
	Rev: CACAAAATTGTCATTCAAGGCAAT			

*The primers were designed for the sequences retrieved from the *C. gigas* genome (Zhang et al., 2012).*

### Statistical Analysis

Normality and homogeneity of variance were tested for residuals and predicted values for all data. Average values from the last time points (130–150 min) were calculated for TER, TEP, and SCC to obtain stable values of the parameters. The effect of salinity on TER, TEP and the transfer of radiolabelled Ca^2+^ as well as on the hemolymph ion concentration was assessed with an Independent Samples *t*-test. The effect of salinity on SCC was assessed with a non-parametric Mann–Whitney *U* test. The statistical analyses for electrophysiology data were performed using IBM SPSS 22 (SPSS Inc., Chicago, IL, United States). A *P*-value of ≤0.05 was regarded as significant. Statistical differences in mRNA expression between salinities (14 vs. 28) were analyzed using an unpaired *t*-test (two-tailed). *P* values < 0.001 (^∗∗∗^), 0.01 (^∗∗^), and 0.05 (^∗^) were considered as significant. Statistical analysis was performed using GraphPad Prism version 7.0a for MacOSX (GraphPad Software, La Jolla, CA, United States)^5^. All data are presented as mean values ± standard error of the mean.

## Results

### Electrophysiology, Ca^2+^ Transfer and Hemolymph Ion Concentration

**FIGURE 2:**
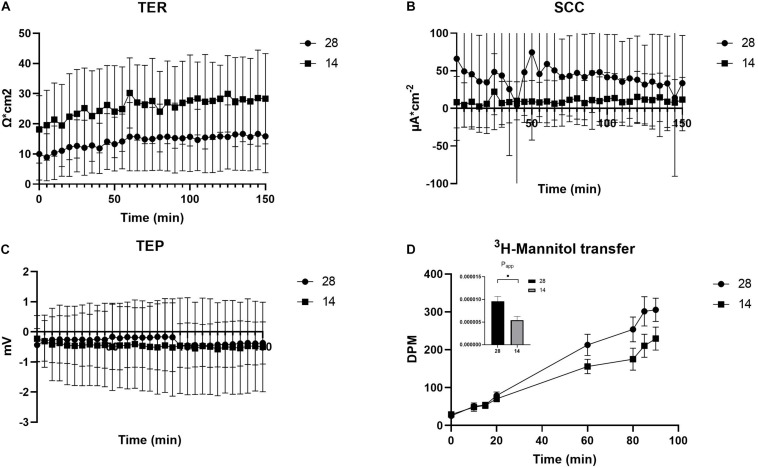
Electrical parameters and apparent permeability of ^3^H-Mannitol of the OME from *C. gigas* exposed to seawater (28) and seawater diluted to 50% (14). The electrical parameters TER **(A)**, SCC **(B)**, and TEP **(C)** were measured for the entire duration of the experiment (60 min for stabilization +90 min for experiments) whereas DPM for ^3^H-Mannitol **(D)** were measured at several time points during the 90 min experimental period. The insertion describes the differences between the *P*_app_ values in the two treatments. Values are shown as the mean ± SEM. Sample sizes, *N*_28_ = 9, *N*_14_ = 10. Significant differences detected by a *t*-test are indicated by (^∗^) *P* < 0.01.

**FIGURE 3:**
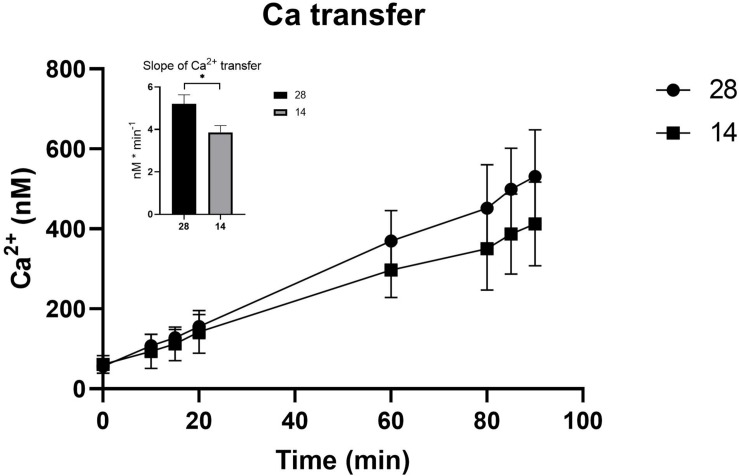
^45^Ca-transfer across the OME from *C. gigas* exposed to seawater (28) and seawater diluted to 50% (14) during the 90 min experimental period. The insertion describes the differences between the transfer slopes (nM ⋅ min^– 1^) in the two treatments. Values are shown as the mean ± SEM. Sample sizes, *N*_28_ = 9, *N*_14_ = 10. Significant differences detected by a *t*-test are indicated by (^∗^) *P* < 0.05.

The electrical parameters TER, TEP, and SCC were monitored throughout the experiment to measure paracellular shunt resistance (TER), net ion distribution (TEP) and active ion transport (SCC) of the *C. gigas* OME. TER showed a tendency toward increased values, 28.1 ± 4.80 Ω ⋅ cm^2^ (average value from time points 130–150 min, *N* = 10), when exposed to a salinity of 14 as compared to oysters in 28 (20.1 ± 3.87 Ω ⋅ cm^2^; *N* = 9, *P* = 0.071, Figure 2A). The SCC showed a tendency toward decreased values when exposed to salinity 14 (10.6 ± 10.9 μA ⋅ cm^–2^, *N* = 10, vs. 41.7 ± 22.2 μA ⋅ cm^–2^, *N* = 9; *P* = 0.315, Figure 2B). The TEP was maintained at the same level irrespective of the salinity of the external environment (Figure 2C). The apparent permeability (*P*_app_) for the hydrophilic marker mannitol, that was a proxy for the passive, paracellular pathway, was higher in salinity 28 (9.58 ⋅ 10^–6^ ± 1.06 ⋅ 10^–6^, *N* = 9) compared to 14 (5.41 ⋅ 10^–6^ ± 8.59 ⋅ 10^–7^, *N* = 10) (*P* = 0.003) (Figure 2D). Ca^2+^ transfer across the OME was higher in salinity 28, 498 ± 37.1 nM/min (*N* = 9), compared to 14, 393 ± 36.1 nM/min (*N* = 10), by 21% (*P* = 0.021) (Figure 3).

Hemolymph Ca^2+^ concentrations decreased from 5.87 ± 0.19 mM to 3.91 ± 0.17 (*P* < 0.001), a 33.4% drop, in oysters maintained in salinity 14 compared to 28. Similarly the concentrations of Na^+^, K^+^, and Cl^–^ decreased by 46.5% (28: 529.6 ± 24.3; 14: 283.6 ± 21.6), 22.3% (28: 11.6 ± 0.39; 14: 9.01 ± 0.62), and 54.3% (28: 421.7 ± 16.5; 14: 192.6 ± 19.8), respectively.

### Ca^2+^ Transporters and Channels in Oyster and Other Mollusks

Searches in the *C. gigas* genome identified three types of Ca^2+^-ATPases: PMCA, SERCA and the secretory pathway Ca^2+^-ATPase (SPCA). Three sequence hits were also retrieved for VGCCs and the genes identified shared the highest sequence similarity with the voltage-gated L-type calcium channel, a voltage-gated P-type channel and a voltage-gated T-type channel. A single *C. gigas* NKA subunit α gene and five putative NCX genes were identified.

Comparative analysis of the *C. gigas* genes with other mollusks identified single genes for different Ca^2+^-ATPase families in two other oysters, *P. fucata* and *C. virginica*, and also in other mollusks, *M. galloprovincialis*, *T. squamosa*, *Mizuhopecten yessoensis*, *Hyriopsis cumingii*, *L. gigantea*, and *O. bimaculoides* as well as in the annelid and brachiopod representatives. The exception was SERCA, for which three different sequences were obtained in *P. fucata* and four putative genes were retrieved from the leech genome. The SPCA members were less represented in bivalves and were only identified in *C. virginica* and in the scallop, *M. yessoensis*. Single genes for NKA were also retrieved from other mollusks while annelids and brachiopods seemed to have multiple members. Multiple NCX genes were found in the mantle transcriptome of the *M. galloprovincialis* as well as in other species suggesting that multiple members of this family exist in mollusks. No NCX representative was identified in annelids but a gene was detected in the brachiopod genome. For the VGCCs a similar number of genes was found in *C. gigas* and other mollusks but in the annelids several additional members seem to exist.

### Phylogeny of Mollusca Ca^2+^ Transporters and Channels

Phylogenetic analysis (using both ML and BI methods) of Ca^2+^ transporters and channels from *C. gigas* and other mollusks and lophotrocozoan species confirmed their identity and revealed that each protein family shared common ancestral origin with the vertebrate homologs. In general, when multiple genes were identified in a species, they seemed to have arisen by lineage or species-specific duplication events. The *C. gigas* sequences tended to cluster with homologs from *C. virginica* and *P. fucata* (evolutionary close species) and other bivalves (the scallop and the mussel species).

**FIGURE 4:**
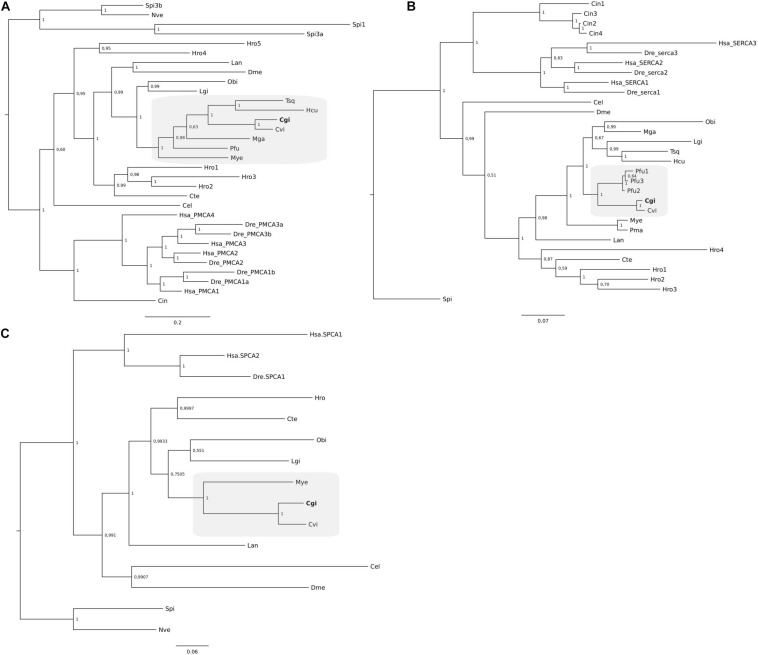
Phylogenetic analysis of SERCA **(A)**, PMCA **(B)**, and SPCA **(C)** from *C. gigas* and other metazoans. The tree was built using the BI method and posterior probability values of the branches are shown. Gene family trees built with the ML method are available as Supplementary Figure S1. The description of sequence abbreviations and accession numbers are presented in Supplementary Table S1. The clusters containing the *C. gigas* sequences are boxed in gray and the *C. gigas* (Cgi) sequences are highlighted in bold. Trees were rooted with the cnidarian sequences.

Phylogenetic analysis of Ca^2+^-ATPases from *C. gigas* together with other mollusk Ca^2+^-ATPases confirmed that a single gene and transcript for the three different members exist (Figures 4A–C). Clustering of the three *P. fucata* SERCA (Figure 4B) sequences revealed that they are highly similar in sequence and only differed at the C-terminus and are probably alternative transcripts of a single gene. Grouping of the genes from annelids and the brachiopod suggests that the multiple members identified arose from species-specific events (Figures 4A–C).

**FIGURE 5:**
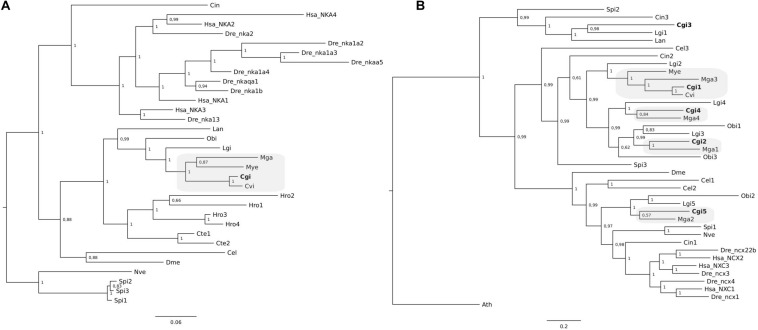
Phylogenetic analysis of NKA **(A)** and NCX **(B)** from *C. gigas* and other metazoans. The tree was built using the BI method and posterior probability values of the branches are shown. Gene family trees built with the ML method are available as Supplementary Figure S2. The description of sequence abbreviations and accession numbers are in Supplementary Table S1. The *C. gigas* sequences are highlighted in bold. The NKA tree was rooted with the cnidarian sequences and the NCX tree with a plant (*Arabidopsis thaliana*) sequence.

**FIGURE 6:**
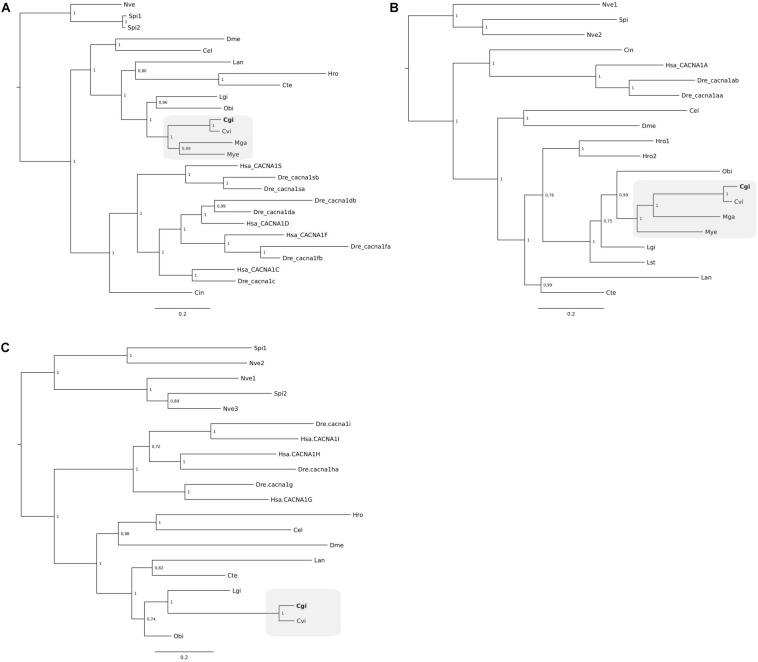
Phylogenetic analysis of the voltage gated calcium channels L-type **(A)**, P-type **(B)**, and T-type **(C)** of *C. gigas* and other metazoans. The tree was built using the BI method and posterior probability values of the branches are shown. Gene family trees built with the ML method are available as Supplementary Figure S3. The description of sequence abbreviations and accession numbers are in Supplementary Table S1. The clusters containing the *C. gigas* sequences are boxed in gray and the *C. gigas* (Cgi) sequences are highlighted in bold. Trees were rooted with the cnidarian sequences.

Clustering of the *C. gigas* and other mollusk NKA gene members suggest that a single NKA gene exists in *C. gigas* and other bivalves but that in annelids and brachiopods multiple genes exist (Figure 5A). Similarly, the three VGCC trees also indicate that single gene copies for Ca channels are present in *C. gigas* and other lophotrochozoans (Figure 6). For the NCX tree clustering of the sequences indicates that this gene family is highly diverse and that three major NCX clusters exist and in the oyster five putative members of this gene family exist (Figure 5B). These include a subfamily that shares high sequence similarity with the vertebrate NCXs and includes *Cgi5*; a second subfamily clustered *Cgi1*, *Cgi2*, and *Cgi4* and this was a characteristic of most of the mollusks and other lophotrochozoans; and a third clade contained *Cgi3* and a brachiopod sequence. Similarly, in *Ciona* three NCX genes were found, which were distributed between the three *subfamilies*. This suggests that the metazoan NCX members resulted from an earlier gene duplication event that probably occurred before the protostome-deuterostome division, and that mollusks retained members of the three subfamilies while in vertebrates only members of one subfamily were retained. In the vertebrates, human and zebrafish, multiple gene members for Ca^2+^-ATPases, NKA, NCX, and VGCCs exist and sequence clustering indicates that they resulted from gene duplication events that occurred at the base of the vertebrate radiation.

### Gene Expression

**FIGURE 7:**
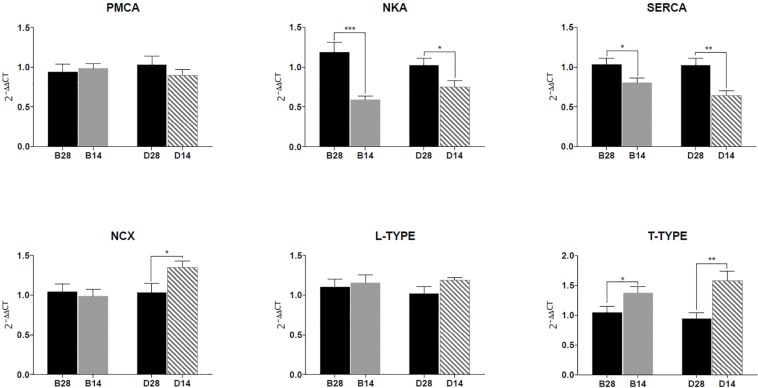
Gene expression of the ion transporters (NKA, PMCA, SERCA, NCX) and calcium channels (L-type and T-type VGCC) in the lower mantle (B) and upper mantle (D) of *C. gigas* exposed for 2 weeks to full seawater (28) or seawater diluted to 50% (14). The results were normalized using the geometric mean of two reference genes, Ef1α and GADPH. The data are presented as the mean ± SEM. Sample sizes *N*_b 14, b 28_ = 10, *N*_d__14_ = 9, *N*_d__28_ = 7. Significant differences detected by an unpaired *t*-test of either the lower (B) or upper (D) mantle from *C. gigas* exposed to seawater at 28 or 14 are indicated as follows, (^∗∗∗^) *P* < 0.001, (^∗∗^) *P* < 0.01 and (^∗^) *P* < 0.05. The mRNA fold changes are described in Supplementary Table S2.

The mRNA expression levels of four different ion transporters, NKA, PMCA, SERCA, NCX, and two calcium channels, L-type and T-type Ca channels were analyzed using qPCR (Figure 7). Both NKA and SERCA expression levels significantly decreased in the mantle of the salinity 14 group compared to the mantle from the salinity 28 group. The expression of T-type VGCC significantly increased in the mantle of the salinity 14 group compared to 28 in both the left and right mantle edge (Figure 7). NCX expression was not significantly modified by salinity in the left mantle but in the right mantle its expression significantly increased in *C. gigas* maintained in 14 (Figure 7). PMCA and L-type VGCC expression was not affected by salinity, nor was there any difference between the two mantle areas analyzed (data not shown).

## Discussion

This study revealed the presence of the gene isoforms of VGCCs, PMCA, and NCX in the mantle tissue of *C. gigas* further suggesting their participation in Ca^2+^ transfer across the OME to the EPS. Adult *C. gigas* exposed to diluted environmental salinity for 2 weeks had decreased transfer of Ca^2+^ across the OME as well as an overall decrease in active ion transport and epithelial permeability. The mRNA expression of the ion transporters, NKA and SERCA decreased in the mantle of *C. gigas* exposed to lower salinity (14) while T-type VGCC and NCX expression increased. The changes in the expression of the ion transporters and channels affected by the decrease in the environmental salinity suggest that the OME cells aim to maintain a high enough intracellular Ca^2+^ concentration for sufficient Ca^2+^ flow for the shell formation area. However, as shown by the decreased Ca^2+^ transfer across the OME, the compensatory mechanisms were insufficient. Although physiological functions of oysters, such as osmoregulation, may adapt to changes in salinity, in the long term the resulting changes in ion transfer could lead to decreased shell mineralization.

The decrease in the Ca^2+^ transfer across the *C. gigas* OME in low salinity could be caused either by a decrease in the transcellular transfer of Ca^2+^ or by a reduction in the paracellular transfer between the OME cells. The active transport of ions measured as SCC also decreased in the 14 salinity which could be due to a decrease in active Ca^2+^ transport through e.g., PMCA and/or NCX. There was, however, no change in PMCA mRNA expression in the 14 salinity. The mRNA expression of the other potential active Ca^2+^-transporter, NCX, increased in the right mantle from *C. gigas* exposed to low salinity, which suggests that the reduction in Ca^2+^ transfer was unlikely to be due to changes in NCX expression. The decrease in the active transport of ions could also be due to a decrease in NKA activity, since NKA maintains the cell membrane potential and establishes an electrochemical gradient for Na^+^ directed into the cell. The reduction in NKA α-subunit mRNA expression in the mantle from *C. gigas* exposed to reduced salinity of 14 supports this notion. However, changes in mRNA expression does not always reflect changes in protein expression since posttranslational modifications as well as protein degradation and synthesis rate can affect protein expression after the gene transcription (Greenbaum et al., 2003). Additionally, a single time point at the end of the 2-week experimental period was measured and although the mRNA expression levels were changed, this does not provide information about the changes occurring in protein activity.

Bivalves are osmoconformers, with hemolymph osmolality following that of the environment (McFarland et al., 2013; Lin et al., 2016). When faced with hypo-osmotic conditions, epithelia tighten up to decrease the leakage of water into the tissues (Shumway, 1977b; Fuhrmann et al., 2018). This may result in a decreased permeability of ions and charged molecules which can be measured as an increased electrical epithelial resistance, TER. A lowered epithelial permeability for water would further result in a decrease in the paracellular transfer of hydrophilic and small molecules across the OME, which can be measured using ^3^H-Mannitol (*P*_app_). The present study shows that in common with other bivalves in *C. gigas* the epithelia are tightened after exposure to a low salinity environment as TER increased and *P*_app_ decreased after acclimation to reduced salinity (14). Connecting back to the Ca^2+^ transfer across the OME, approximately 40% of this occurs via the paracellular pathway (Sillanpää et al., 2018) suggesting that the decrease in the permeability could also contribute to the decrease in the total Ca^2+^ transfer in the oysters exposed to salinity 14.

In humans, four NKA α-subunit isoforms exist, but in *C. gigas* and other bivalves only a single copy was identified. In general, marine invertebrates only have one or two NKA-α isoforms, suggesting that the multiple vertebrate isoforms arose from a later gene duplication event (Sáez et al., 2009; Lind et al., 2013; Thabet et al., 2016). In addition to its role as a house keeping ATPase in all cells, NKA participates in the osmoregulation of marine organisms. Reduction in environmental salinity has been shown to decrease the mantle NKA activity in multiple bivalve species such as *Meretrix lusoria* and *M. galloprovincialis* as well as in the abalone *Haliotis discus hannai* (Borgatti et al., 2003; Lin et al., 2016; Jia and Liu, 2018). However, in some species hypo-osmotic conditions did not affect the NKA mRNA expression in the mantle (Paganini et al., 2010), gills (Lin et al., 2016) or whole body (Willmer, 1978; Peng et al., 2019) and in other cases increased it. Interestingly, when either up- or down-regulated, the need to maintain an electrochemical gradient across the cell membrane is suggested to explain the observed changes in NKA expression. The NKA mRNA expression in the present study may be down-regulated as a direct consequence of lowered Na^+^ concentration in the hemolymph, as this would decrease the passive gradient of both ions across the membrane and thus fewer NKA proteins would be needed to maintain the intracellular Na^+^ concentrations. However, the effects seem to be dependent on species and/or tissue used for extraction, or the experimental set-up.

According to Sillanpää et al. (2018), Ca^2+^ is suggested to enter OME cells through VGCCs. Multiple VGCC genes were found in the *C. gigas* genome, which were homologs of the human L-, P-, and T- type Ca channels. While the L-type and P-type VGCCs belong to the high voltage-activated channels, the T-type (transient) channels are characterized by their fast inactivation and low voltage threshold (Snutch et al., 2000–2013). All types have previously been identified in other mollusks although their exact function remains unknown (Kits and Mansvelder, 1996). In the bivalve *P. fucata*, L-type channels are present in the gills and mantle fold epithelial cells suggesting they have a role in Ca^2+^ uptake from the environment (Fan et al., 2007b). This is further supported by observations that during larval shell development in *M. edulis* Ca channel expression and SERCA expression are up-regulated suggesting that they respond to an increased need for Ca^2+^ for shell mineralization (Ramesh et al., 2019). Changes in the expression of ion channels and transporters may also be related to the adaption to the new environmental salinity. When cells are exposed to hypo-osmotic medium, they swell due to the inflow of water through the cell membrane. To counteract this swelling and to return back to the original volume, cells need to excrete organic and/or inorganic osmolytes to gain equilibrium with the environmental osmolality (Pierce and Greenberg, 1973). Ion extrusion has been suggested to be part of volume regulation and to precede the excretion of the organic osmolytes which make up the main part of the volume regulation in invertebrates (McCarty and O’Neil, 1992; Silva and Wright, 1994). As observed in the present study VGCCs were up-regulated in *C. gigas* and *C. virginica* reared in low salinity, possibly due to their function in cell volume regulation (Meng et al., 2013; Eierman and Hare, 2014).

Even though the model by Sillanpää et al. (2018) did not include SERCA, the present study suggests that they have an important role in the Ca^2+^ metabolism of the mantle cells. Humans possess three SERCA isoforms, but only one SERCA gene has been found in most invertebrates, although this gene can undergo alternative splicing to produce multiple tissue specific proteins (Altshuler et al., 2012). In *P. fucata* for example, three SERCA isoforms that share high sequence similarity (97–99%) and differ only at the C-terminus are alternatively spliced transcripts of a single gene (Fan et al., 2007a). The *P. fucata* SERCA has a tissue specific expression pattern with one isoform being expressed predominantly in the mantle and gill tissues (Fan et al., 2007a). Only a single SERCA isoform was expressed in the mantle of *C. gigas* and it was responsive to changes in environmental ion concentration suggesting it has an important role in Ca^2+^ metabolism and possibly shell mineralization.

The transfer of Ca^2+^ from the OME cells to the EPS was suggested to be executed via an apical PMCA and possibly an apical NCX (Sillanpää et al., 2018). In *C. gigas*, as in other bivalves, a single PMCA gene exists, which can generate multiple isoforms. PMCA is a high affinity, low capacity transporter in contrast to NCX, which is a low affinity high capacity transporter (Blaustein et al., 2002). PMCA can respond rapidly to changes in intracellular Ca^2+^ concentrations and thus finely regulate intracellular Ca^2+^ levels. However, it may become saturated when high levels of Ca^2+^ have to be transported to the EPS (Blaustein and Lederer, 1999). This suggests that PMCA is probably important for the continuous transfer of Ca^2+^ to the extrapallial fluid (EPF). In contrast, NCX, together with ER and other Ca storing compartments, may be important during periods of bulk transfer of Ca^2+^ such as occur during shell biomineralisation, especially when the inflow or outflow of Ca^2+^ are changed. The subcellular location of PMCA, and its likely function can be deduced from its amino acid sequence and the protein splice variants. An insertion larger than 30 amino acids at splice site A compared to the human PMCA 2 has been suggested to imply an apical location (Enyedi and Strehler, 2011). The *C. gigas* PMCA found from the genome contained an insertion of 39 amino acids in the homolog region of splice site A of human PMCA, which implies it may have an apical location (Supplementary Figure S4, Enyedi and Strehler, 2011; Zhang et al., 2012). The 39 amino acid insertion in *C. gigas* PMCA is similar to that reported in *T. squamosa* (Asp285-Asn320), which has been shown by immunofluorescent microscopy to have an aplical location (Ip et al., 2017). These findings support the model of Sillanpää et al. (2018) where the functional studies indicated an apical localization of PMCA.

Multiple NCX genes were found in the *C. gigas* genome, although only one resembles the vertebrate isoforms. Some invertebrates contain multiple genes with similarity to NCX, but only one gene product functions as a true Na^+^/Ca^2+^-exchanger i.e., a membrane protein executing the exchange of Ca^2+^ for a Na^+^ and driven by the electrochemical gradient across the cell membrane (On et al., 2008). The other genes annotated as NCX share similar motifs to the functional NCX but lack some critical amino acid sequences for the Na^+^/Ca^2+^-exchanging function. Previously, the presence of a NCX in *C. gigas* could not be directly verified using inhibitors against the vertebrate NCX, but a decrease in the Ca^2+^ transfer after inhibition of the NKA provided indirect evidence for the participation of NCX in the Ca^2+^ transfer across the OME (Sillanpää et al., 2018). In this study, we show that the NCX is expressed in the mantle tissue of *C. gigas* and could thus potentially participate in the Ca^2+^ transporting mechanisms necessary for shell calcification. NCX, together with PMCA are proposed to participate in calcification in larval *M. edulis* (Ramesh et al., 2019) and coral *Acropora yongei* (Barron et al., 2018).

Taken together, the results from the electrophysiological and permeability measurements show that both active and passive ion transport across the OME decreases in *C. gigas* maintained in diluted seawater when compared to the salinity 28. However, the mRNA expression data indicates that the response of the individual transporters and channels is not uniform. Even though the mRNA expression of transporters was measured in the whole mantle tissue instead of solely the OME, the results suggest that when exposed to reduced salinity the oysters aim to maintain stable Ca^2+^ metabolism despite the lower water and hemolymph ion concentration. The intracellular Ca^2+^ concentration in the cells is physiologically well regulated and maintained at a constant level. Ca^2+^ enters the mantle cells through VGCCs down its electrochemical gradient in a passive and concentration dependent manner. Therefore, if the extracellular Ca^2+^ concentration varies, the cell Ca^2+^ uptake, release and sequestration need to be adjusted accordingly. In the current study, the increase in mRNA expression of the T-type VGCCs in the salinity 14-group suggests that the mantle cells adjust to reduced extracellular Ca^2+^ concentrations by up-regulating systems that promote Ca^2+^ entry into cells. The decreased mRNA expression of SERCA in low salinity may reflect an additional compensatory mechanism, to maintain Ca^2+^ levels in the cytosol through less sequestration of Ca^2+^ into the endoplasmic reticulum (ER). From the perspective of shell biomineralisation, an increase in the expression or activity of Ca^2+^ transporters and channels in dilute seawater would contribute to maintain a stable Ca^2+^ transfer rate to the shell building area. In other words, the oysters may compensate for decreased environmental Ca^2+^ concentrations by decreasing Ca^2+^ sequestration in the ER and keeping the amount of available Ca^2+^ in the cytosol ready for transport to the EPF stable. As NCX often responds to changes in the cellular in/outflow of Ca, an increase in its expression might be coupled to changed intracellular Ca^2+^ uptake and sequestration (Ottolia et al., 2013; Seth et al., 2013). The functional relevance of the changes in VGCC and SERCA expression in the mantle of *C. gigas* maintained in a reduced salinity environment (14) may be associated with a shift in the intracellular Ca^2+^ balance, which promotes increased extrusion of Ca^2+^ from the mantle cells. However, it cannot be excluded that the up-regulation of NCX in the oysters acclimatized to salinity 14 may be a results of a decreased Na^+^ concentration in the hemolymph. Up-regulation of NCX in a low Na^+^ environment would be a means by which to maintain the same rate of Na^+^/Ca^2+^ exchange. To summarize, the decrease detected in the total transfer of Ca^2+^ across the OME implies that the OME cells were not able to maintain a stable rate of Ca^2+^ transfer across the cell membranes, despite an up-regulation of T-type VGCC and NCX.

Although it was not considered in this study, hypo-osmotic conditions have previously been shown to affect many Ca-binding and -regulating proteins in *C. gigas* (Zhao et al., 2012) and probably play an important role in the regulation of intracellular Ca^2+^ concentrations. Prolonged salinity stress might lead to activation of the immune system, which also tends to increase cytosolic Ca^2+^ concentrations (Zhao et al., 2012) and might thus affect Ca^2+^ sequestration and extrusion. Stress from exposure to suboptimal salinity might also suppress biomineralisation to save energy for other essential physiological processes. The significant suppression in total Ca^2+^ transfer across the OME of *C. gigas* maintained in diluted seawater in the present study indicates that prolonged periods in low salinity have the potential to modify shell calcification and maintenance. Changes in the environmental salinity and ion concentration could also potentially affect the carbonate metabolism in calcifying organisms. The expression of a putative HCO_3_^–^ transporter was up-regulated in larval *M. edulis* raised in limiting carbonate concentrations (Ramesh et al., 2019) suggesting that carbonate availability is also affected by the environmental conditions. Combined with other environmental stressors such as increased pCO_2_, temperature or decreased pH, the effects of salinity on carbonate metabolism can be even more pronounced (Waldbusser et al., 2011; Dickinson et al., 2012). The uptake, transport and metabolic production of HCO_3_^–^/CO_3_^2–^ as well as the interactions with Ca^2+^ transport therefore require further attention to elucidate the full picture of how ion transport for shell growth is affected by environmental conditions.

## Conclusion

In conclusion, the results of the present study support the model previously proposed by Sillanpää et al. (2018) by revealing that VGCCs, PMCA, and NCX are expressed and regulated in the mantle tissue of *C. gigas* and have a potential role in Ca^2+^ transfer for biomineralisation. In addition, it was demonstrated that the exposure of *C. gigas* to reduced environmental salinity (14) decreased active ion transport and Ca^2+^ transfer across the OME. The associated change in the mRNA expression of T-type VGCC, SERCA, and NCX in the mantle of *C. gigas* acclimated to salinity 14 suggests that they have the ability to adjust ion regulation when environmental conditions change but that the Ca^2+^ available for shell growth is reduced. Taken together, the results indicate that the environmental fluctuations in costal water salinities, possibly emphasized as a consequence of climate change, may impact the ion transport by the mantle and thus shell calcification in *C. gigas*.

## Data Availability Statement

The datasets generated for this study are available on request to the corresponding author.

## Author Contributions

JS was the lead author, performed the physiological experiments, data analysis, and interpretation of the data, and participated in the design of the study. JC ran the phylogenetic analysis and contributed with ideas and planning of the experiment. RF and LA did the gene expression data analysis as well as contributed with ideas and planning of the experiment. DP and KS designed and coordinated the study as well as contributed in discussion and interpretation of the data. All authors participated in the preparation of the manuscript.

## Conflict of Interest

The authors declare that the research was conducted in the absence of any commercial or financial relationships that could be construed as a potential conflict of interest.

## Footnotes

1 http://ensemblgenomes.org

2 https://blast.ncbi.nlm.nih.gov/Blast.cgi

3 http://www.ensembl.org

4 https://genome.jgi.doe.gov/Nemve1/Nemve1.home.html

5 www.graphpad.com

## Supplementary Material

FIGURE S1 Phylogenetic analysis of SERCA **(A)**, PMCA **(B)**, and SPCA **(C)** from the *C. gigas* and other metazoans. The tree was built using PhyML software from the ATGC platform. Branch support bootstrap values >50% only are shown. The description of sequence abbreviations and accession numbers are in Supplementary Table S1. The clusters containing the *C. gigas* sequences are boxed in gray and the *C. gigas* (Cgi) sequences are highlighted in bold. Trees were rooted with the cnidarian sequences.

FIGURE S2 Phylogenetic analysis of NCX **(A)** and NKA **(B)** from the *C. gigas* and other metazoans. The tree was built using PhyML software from the ATGC platform. Branch support bootstrap values >50% only are shown. The description of sequence abbreviations and accession numbers are in Supplementary Table S1. The clusters containing the *C. gigas* sequences are boxed in gray and the *C. gigas* (Cgi) sequences are highlighted in bold. The NKA tree was rooted with the cnidarian sequences and the NCX tree with a plant, *Arabidopsis thaliana* sequence.

FIGURE S3 Phylogenetic analysis of L-type **(A)**, P-type **(B)**, and T-type **(C)** voltage gated calcium channels from the *C. gigas* and other metazoans. The tree was built using PhyML software from the ATGC platform. Branch support bootstrap values >50% only are shown. The description of sequence abbreviations and accession numbers are in Supplementary Table S1. The clusters containing the *C. gigas* sequences are boxed in gray and the *C. gigas* (Cgi) sequences are highlighted in bold. Trees were rooted with the cnidarian (Nve, Spi) sequences.

FIGURE S4 *C. gigas* and human PMCA sequence.

TABLE S1 Accession numbers and the full description of the sequence abbreviations used in the phylogenetic analysis.

TABLE S2 Fold changes of the mRNA expression measurements.
