# Supplementary figures and images for: Dilution of Seawater Affects the Ca2 + Transport in the Outer Mantle Epithelium of Crassostrea gigas
Source: Front Physiol. 2020 Jan 22;11:1. doi: 10.3389/fphys.2020.00001 (PMC6987452; doi:10.3389/fphys.2020.00001)

A

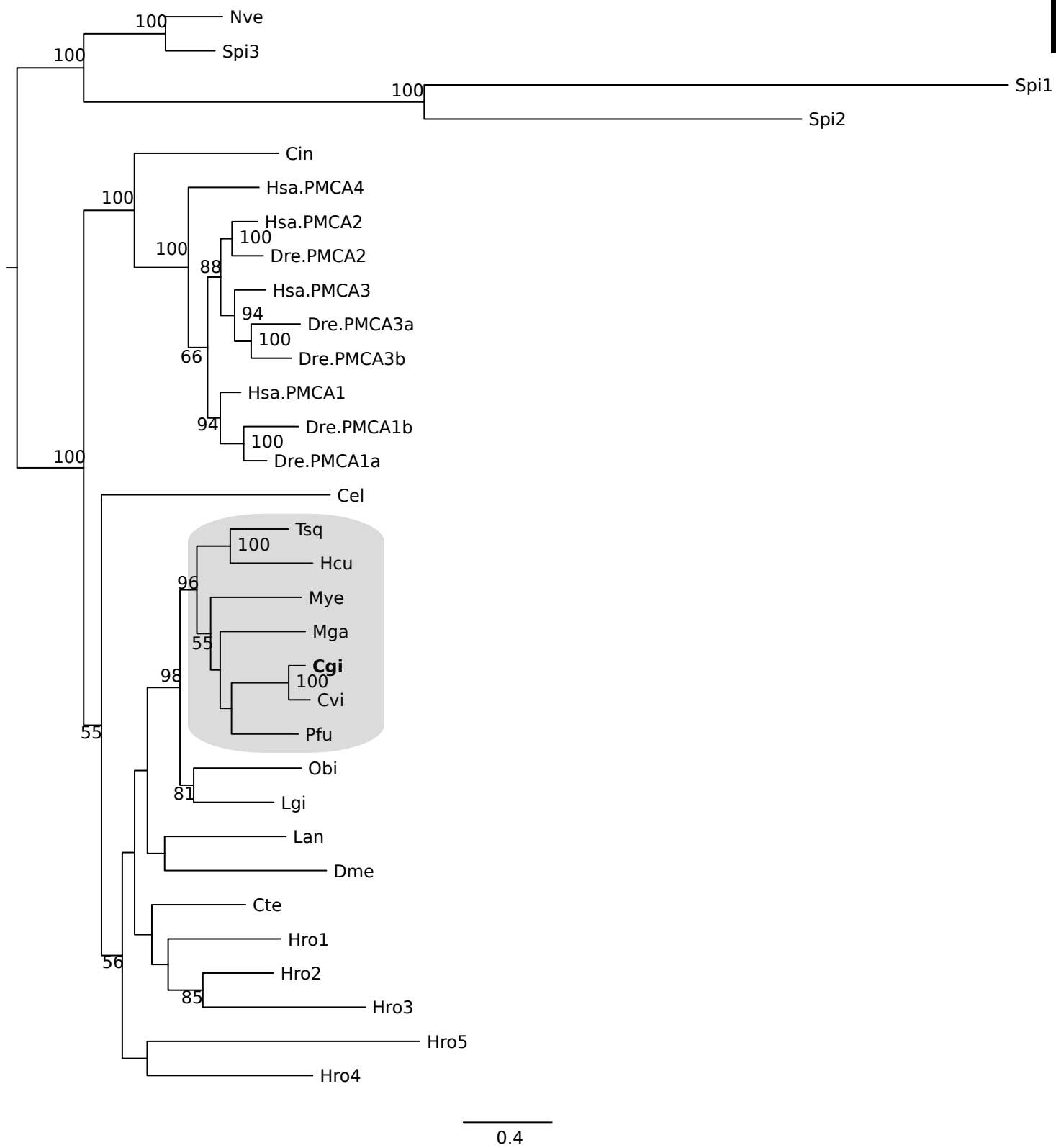

B

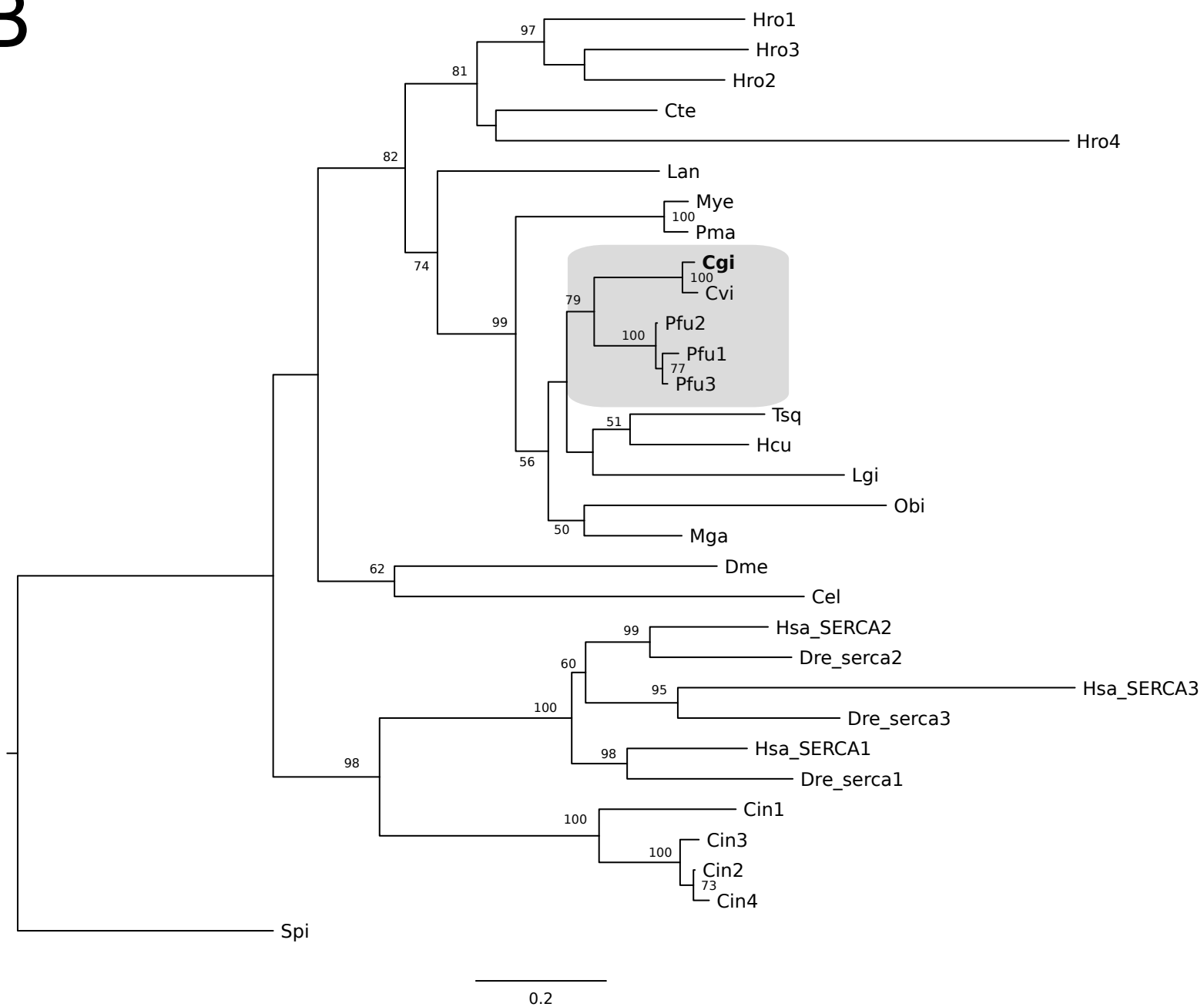

C

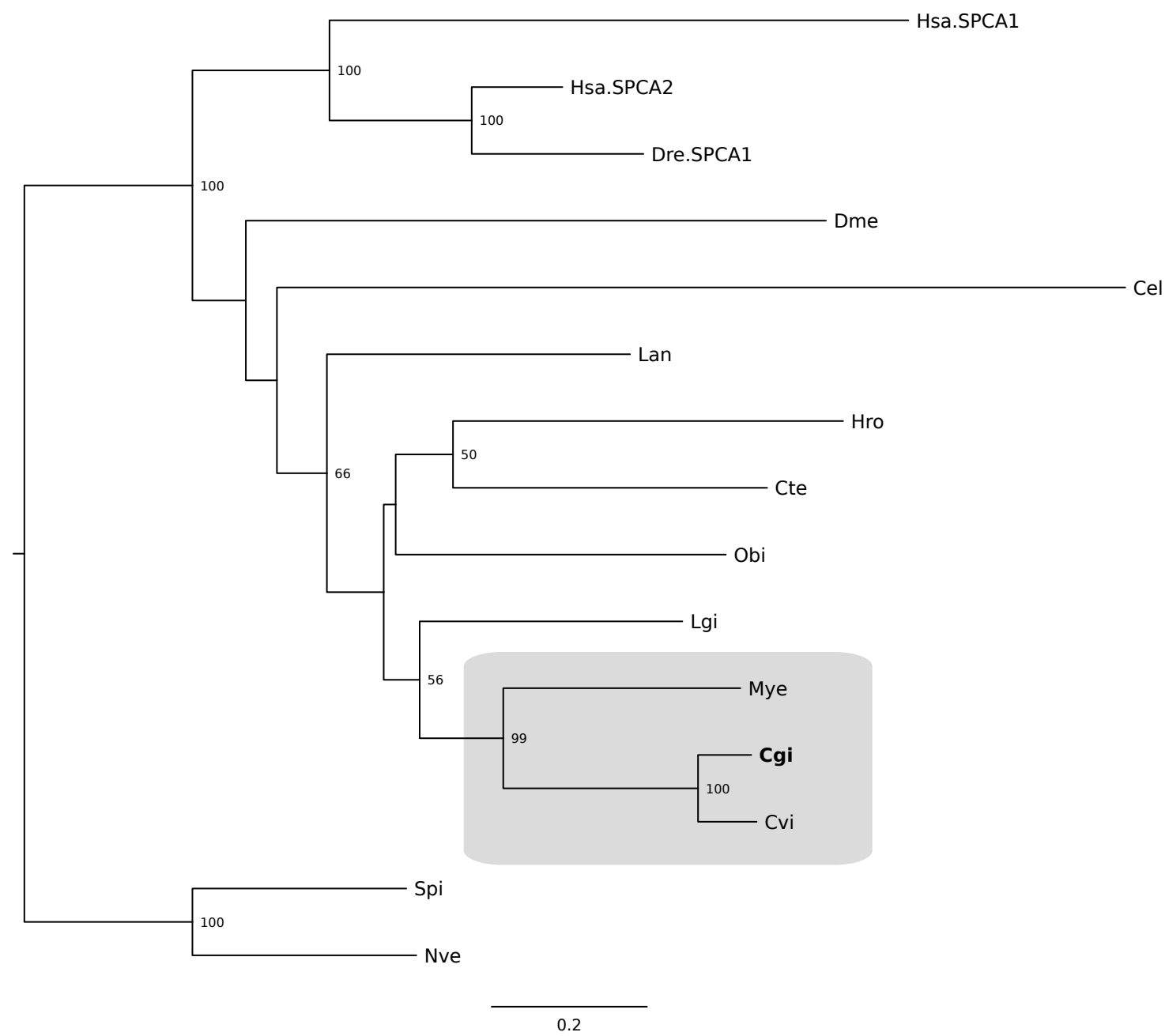

Supplement: FIGURE S1 — Phylogenetic analysis of SERCA (A), PMCA (B), and SPCA (C) from the C. gigas and other metazoans. The tree was built using PhyML software from the ATGC platform. Branch support bootstrap values >50% only are shown. The description of sequence abbreviations and accession numbers are in Supplementary Table S1. The clusters containing the C. gigas sequences are boxed in gray and the C. gigas (Cgi) sequences are highlighted in bold. Trees were rooted with the cnidarian sequences. [file Data_Sheet_1.PDF]

A

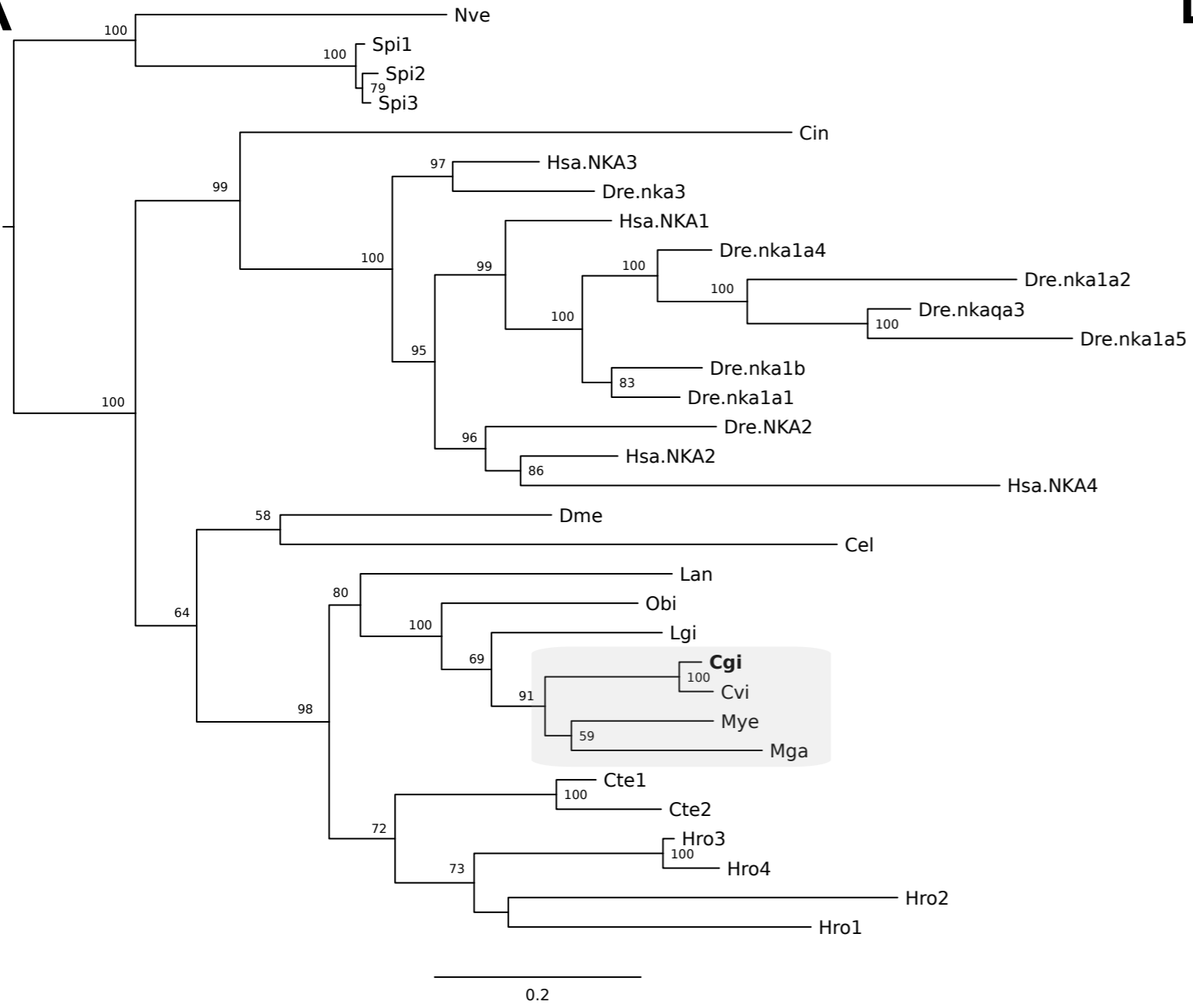

B

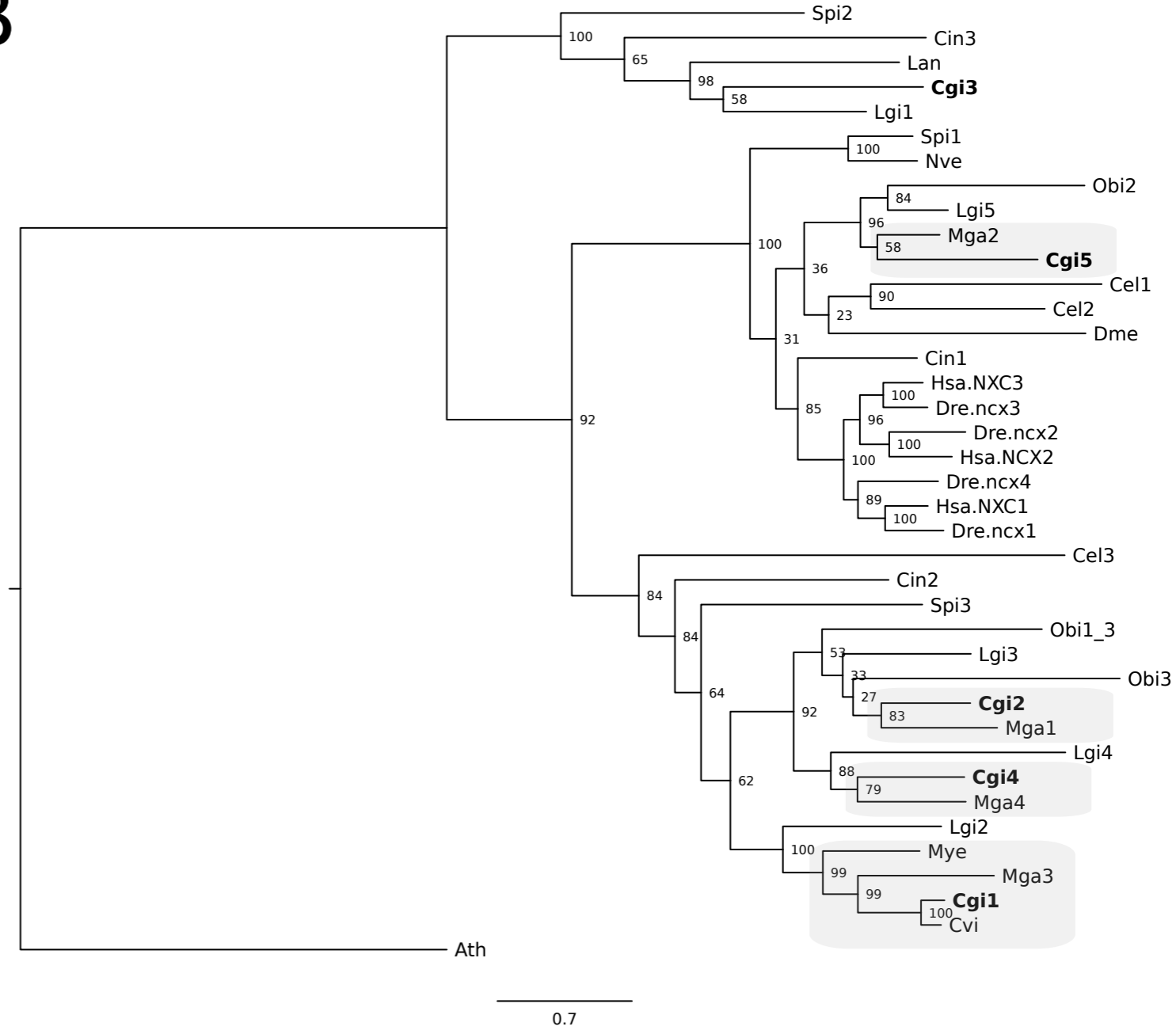

Supplement: FIGURE S2 — Phylogenetic analysis of NCX (A) and NKA (B) from the C. gigas and other metazoans. The tree was built using PhyML software from the ATGC platform. Branch support bootstrap values >50% only are shown. The description of sequence abbreviations and accession numbers are in Supplementary Table S1. The clusters containing the C. gigas sequences are boxed in gray and the C. gigas (Cgi) sequences are highlighted in bold. The NKA tree was rooted with the cnidarian sequences and the NCX tree with a plant, Arabidopsis thaliana sequence. [file Data_Sheet_2.PDF]

A

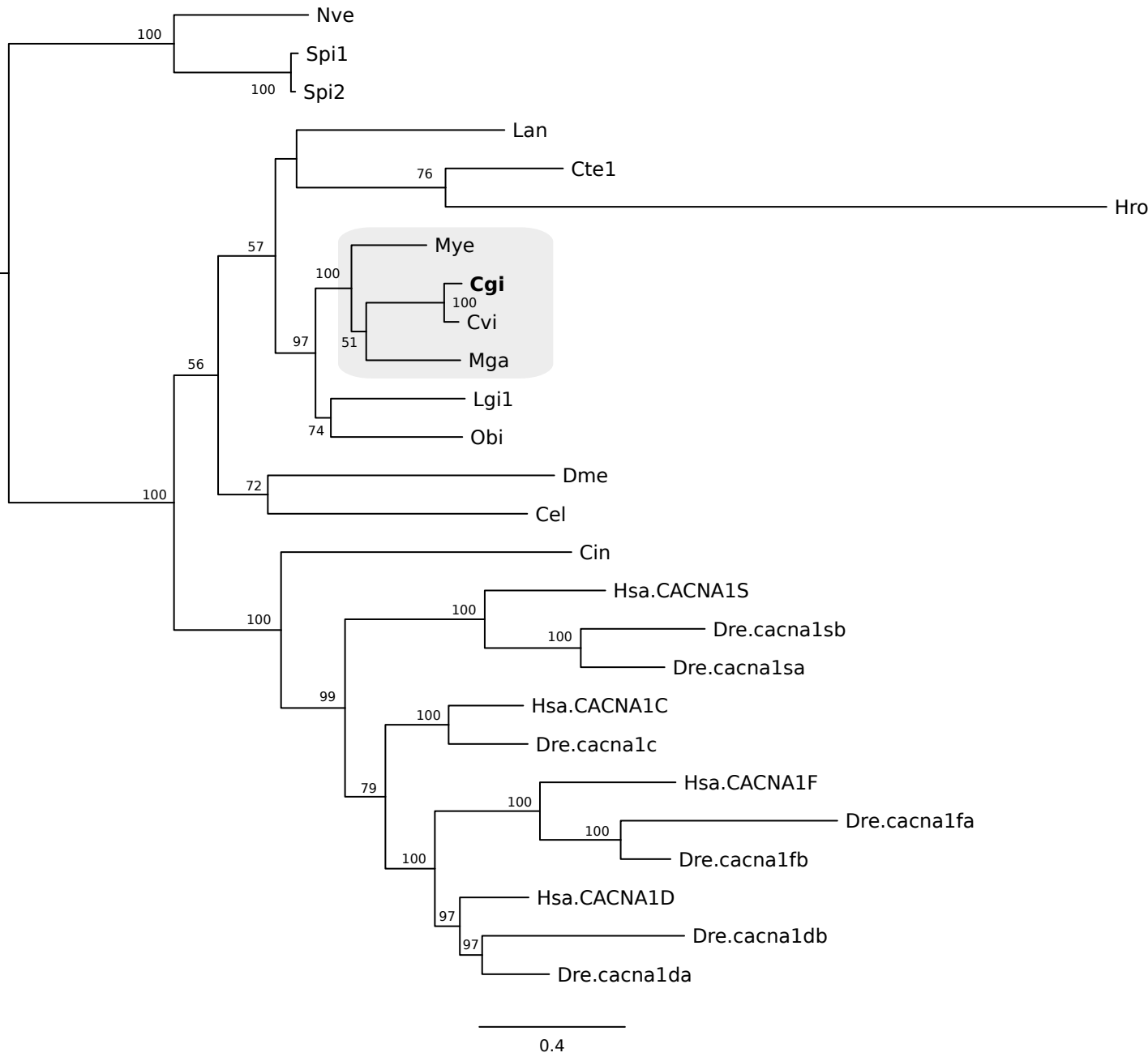

B

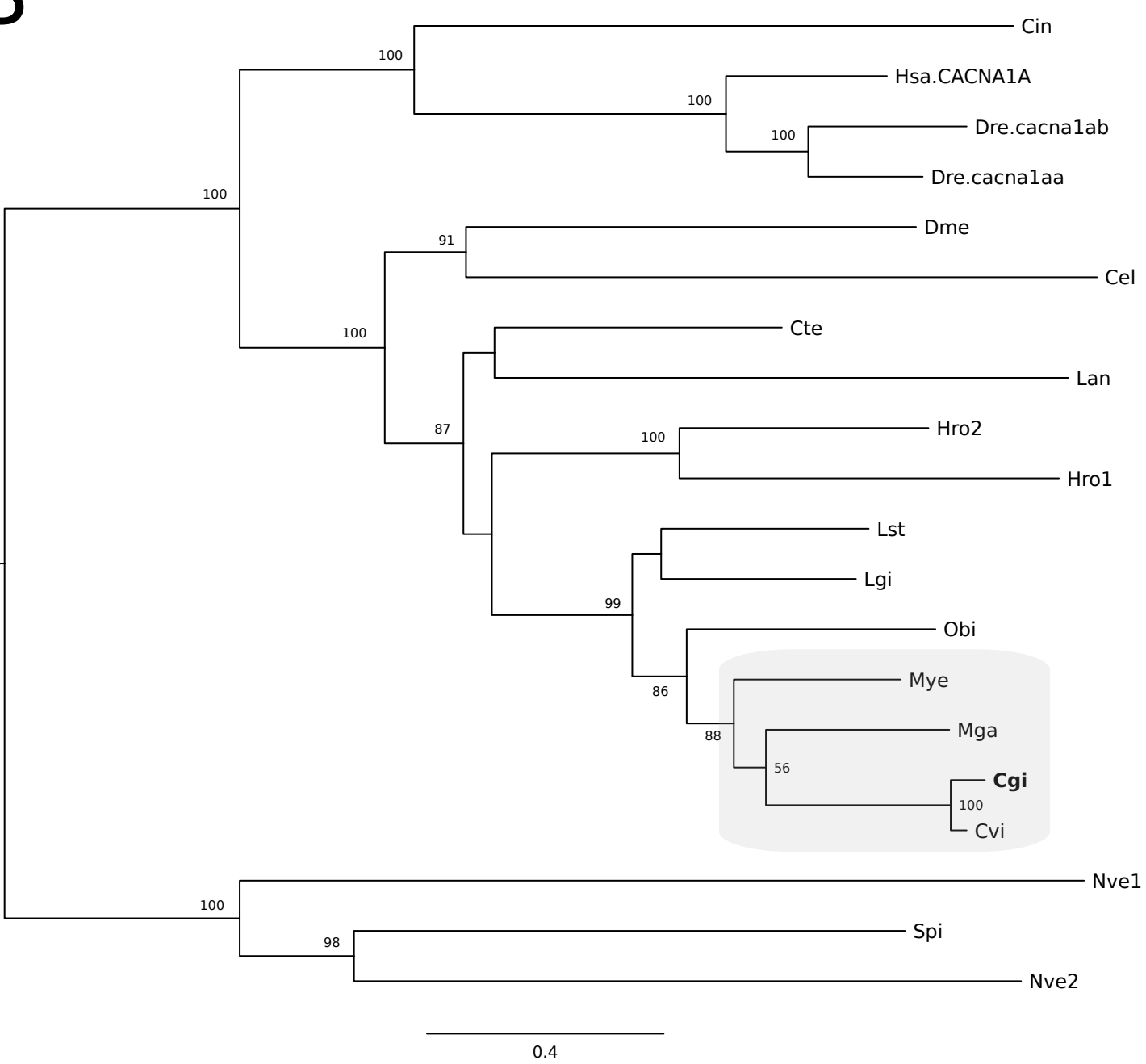

C

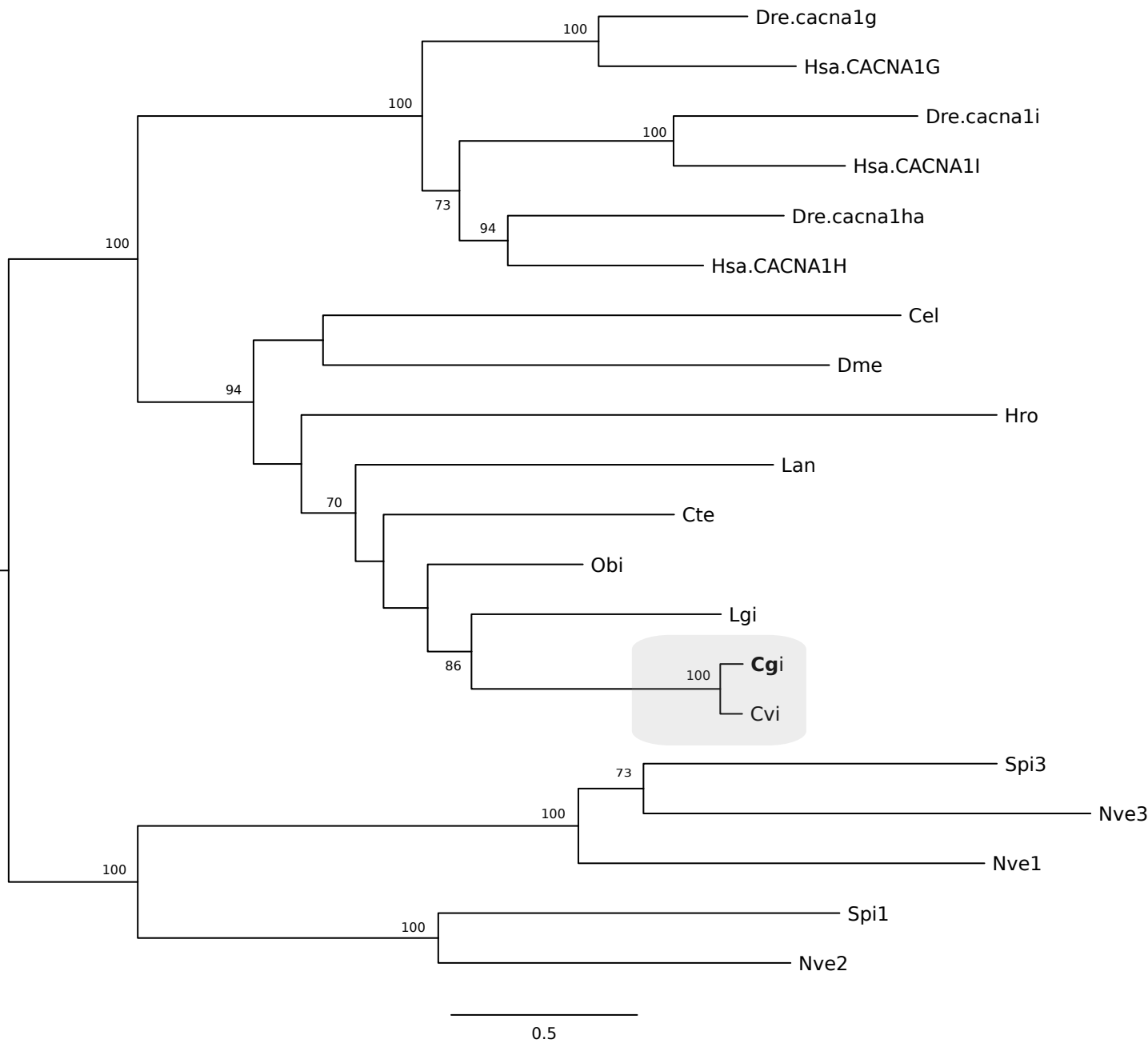

Supplement: FIGURE S3 — Phylogenetic analysis of L-type (A), P-type (B), and T-type (C) voltage gated calcium channels from the C. gigas and other metazoans. The tree was built using PhyML software from the ATGC platform. Branch support bootstrap values >50% only are shown. The description of sequence abbreviations and accession numbers are in Supplementary Table S1. The clusters containing the C. gigas sequences are boxed in gray and the C. gigas (Cgi) sequences are highlighted in bold. Trees were rooted with the cnidarian (Nve, Spi) sequences. [file Data_Sheet_3.PDF]
