# Supplementary material for: Dilution of Seawater Affects the Ca2 + Transport in the Outer Mantle Epithelium of Crassostrea gigas
Source: Front Physiol. 2020 Jan 22;11:1. doi: 10.3389/fphys.2020.00001 (PMC6987452; doi:10.3389/fphys.2020.00001)
Supplement: FIGURE S4 — C. gigas and human PMCA sequence. [file Data_Sheet_4.PDF]

```
Cgi_PMCA3_ : EKTDPMMFSGTHVMEGSGKMLVTAVGVNSQTGIIFALLGASSEEGEKDKKKDKKDKNRLKGEEGNGGTRTNTAGQNTPYIDSIHPVIPTGN--------- :  327

Hsa_PMCA1_ : LDKDPLLLSGTHVMEGSGRMVVTAVGVNSQTGIIFTLLGAGGE-------EEEKKDEKKKEKKNKKQDGA------------------------------ :  316
Hsa_PMCA2_ : VDKDPMLLSGTHVMEGSGRMLVTAVGVNSQTGIIFTLLGAGGEEEEKKDKK------------------------------------------------- :  302
Hsa_PMCA3_ : ADKDPMLLSGTHVMEGSGRMVVTAVGVNSQTGIIFTLLGAGGEEEEKKDKKG------------------------------------------------ :  306

Hsa_PMCA4_ : LDKDPMLLSGTHVMEGSGRMVVTAVGVNSQTGIILTLLGVNEDDEGEKKKKGKKQG-------------------------------------------- :  305
```
