# Supplementary material for: Dilution of Seawater Affects the Ca2 + Transport in the Outer Mantle Epithelium of Crassostrea gigas
Source: Front Physiol. 2020 Jan 22;11:1. doi: 10.3389/fphys.2020.00001 (PMC6987452; doi:10.3389/fphys.2020.00001)
Supplement: TABLE S1 — Accession numbers and the full description of the sequence abbreviations used in the phylogenetic analysis. [file Data_Sheet_5.PDF]

	**B32**	**B16**	**D32**	**D16**
**PMCA**	*0.94±0.10*	*0.98±0.06*	*1.03±0.11*	*0.90±0.07*
**NKA**	*1.19±0.12*	*0.59±0.05*	*1.02±0.09*	*0.75±0.08*
**SERCA**	*1.03±0.08*	*0.80±0.07*	*1.02±0.09*	*0.65±0.05*
**NCX**	*1.04±0.10*	*0.99±0.09*	*1.03±0.11*	*1.34±0.08*
**L-Type**	*1.10±0.10*	*1.15±0.10*	*1.02±0.09*	*1.19±0.03*
**T-Type**	*1.04±0.11*	*1.37±0.10*	*0.94±0.10*	*1.57±0.16*
